# Parasympathetic Tone Changes in Anesthetized Horses after Surgical Stimulation, and Morphine, Ketamine, and Dobutamine Administration

**DOI:** 10.3390/ani12081038

**Published:** 2022-04-15

**Authors:** Patricia Ruíz-López, Juan Morgaz, Setefilla Quirós-Carmona, Rocío Navarrete-Calvo, Juan Manuel Domínguez, Rafael Jesús Gómez-Villamandos, M. M. Granados

**Affiliations:** 1 Department of Surgery and Anesthesia of Domestic Animals, Faculty of Veterinary Medicine, University of Ghent, 9820 Merelbeke, Belgium; 2 Animal Medicine and Surgery Department, Faculty of Veterinary Medicine, University of Córdoba, 14014 Cordoba, Spain; setequica@gmail.com (S.Q.-C.); nacar6@hotmail.com (R.N.-C.); pv2dopej@uco.es (J.M.D.); pv1govir@uco.es (R.J.G.-V.); pv2grmam@uco.es (M.M.G.)

**Keywords:** autonomic nervous system, dobutamine, horses, ketamine, morphine, nociception, parasympathetic tone activity, PTA monitor

## Abstract

**Simple Summary:**

A parasympathetic tone activity (PTA) monitor has been developed similar to the analgesia nociception index (ANI) used in human medicine to evaluate the changes in the autonomic nervous system based on heart rate variability. The autonomic nervous system acts unconsciously and regulates body functions (autonomic response). Examples of autonomic response are decreases of heart rate or/and blood pressure due to an increase of parasympathetic tone activity. It is important to know how stimuli and medication may affect the autonomic nervous system since they can modify heart rate and blood pressure. This study attempts to find how a surgical nociceptive stimulus, along with the administration of medication frequently used in horses, can affect mean parasympathetic tone activity (PTAm) (one of the values of the PTA monitor), heart rate and blood pressure in clinically anesthetized horses. Values of the PTAm, heart rate, and blood pressure were registered before and after surgical incision and after the administration of morphine, ketamine, and dobutamine at defined time points. No changes were found after the incision or the administration of morphine and dobutamine. It seems that only ketamine affects the autonomic nervous system by decreasing PTAm.

**Abstract:**

Autonomic nervous system (ANS) activity can modify cardiovascular parameters in response to nociceptive stimuli or drugs in anesthetized animals. The aim of this study was to determine if a surgical nociceptive stimulus and morphine, ketamine, and dobutamine administration would modify ANS activity observed as a change in the mean parasympathetic tone activity (PTAm) in anesthetized horses. In 20 anesthetized horses, heart rate (HR), mean arterial pressure (MAP), and PTAm were monitored before and 1, 3, and 5 min after surgical incision, and before and 10 min after the administration of morphine (0.2 mg/kg IV). If nystagmus or spontaneous ventilation was observed, ketamine (0.5 mg/kg IV) was given, and the three variables were registered before and 3 and 5 min afterward. If MAP reached ≤62 mmHg, a dobutamine infusion was administered, and the three variables were recorded before and 5 min after starting/increasing the infusion (0.25 μg/kg/min IV every 5 min). The three variables were registered before and 1, 3, and 5 min after a PTAm decrease of ≥20%, HR increase of ≥10%, or MAP increase of ≥20%. The PTAm decreased 3 min after the administration of ketamine and 1 min after a PTA event. The surgical incision, dobutamine, and morphine did not modify PTAm. The absence of changes in ANS activity after the nociceptive stimulus and lack of correlation between PTAm and HR or MAP suggest that PTAm is a poor indicator of sympathetic activation under the study conditions. Ketamine seems to affect ANS activity by decreasing PTAm.

## 1. Introduction

The autonomic nervous system (ANS) plays an important role in modifying heart rate (HR), blood pressure [1], and HR variability (HRV) [2] in response to nociceptive stimuli [2] or the administration of drugs [3]. An increase in parasympathetic nervous system activity decreases HR, consequently increasing the R-R interval and changing the HRV [4]. The sympathovagal balance can be modified during anesthesia by a variety of perioperative factors, including anesthetic drugs, resulting in hemodynamic changes [1]. The HRV shows the influence of parasympathetic and sympathetic systems on the sinoatrial node and, therefore, HR; however, HRV does not represent an absolute level of autonomic activity [5]. Monitors based on HRV analysis as a measure of ANS activity have been developed. The analgesia nociception index (ANI) is used in humans [6], while the parasympathetic tone activity (PTA) monitor (PTA monitor, Metrodoloris, Lille, France) analyzes the HRV of dogs, cats, and horses [7,8,9,10]. The PTA monitor is a non-invasive device that displays two-dimensional values, updated every second, ranging from 0 to 100. The highest value reflects an absence of sympathetic response [7,11]. The mean or average PTA (PTAm) is the mean value obtained during the last 176 s, and the instantaneous PTA is the mean value of the PTA values obtained over the last 54 s [10,12]. The PTA monitor provides a constant quality measurement and a graphic related to respiratory sinus arrhythmia and parasympathetic tone. Considering respiration is related to cardiac vagal changes [13], stable breathing is needed for optimal performance [2].

The PTA monitor reflects nociception-antinociception balance and predicted cardiovascular reactivity, detecting the response to nociceptive stimuli of lower intensities earlier than cardiovascular variables in anesthetized dogs [6,7,9]. It was able to differentiate three drug regimens related to analgesia in pigs [11]. In horses that received a continuous rate infusion (CRI) of fentanyl, the PTAm was higher than in horses that did not receive it during surgery [14]. This monitor also showed lower values of PTAm in horses undergoing colic surgery compared to elective surgeries [9]. Several drugs might affect ANS activity. For example, ketamine [15,16], opioids [17], and dobutamine [18,19] may have sympathomimetic properties. Data regarding the influence of different anesthetic drugs on ANS activity are extrapolated from human medicine. There is a need to elucidate how drugs commonly used for equine anesthesia can affect the ANS activity and interfere with the PTA monitor performance during general anesthesia [9].

The objectives of this study were: (1) to determine if a surgical nociceptive stimulus or drug administration (morphine, ketamine, or dobutamine) would produce changes in ANS activity, as measured by the PTA monitor in anesthetized horses; and (2) to determine if a correlation between PTAm, HR, and mean arterial pressure (MAP) exists during surgical stimulation. We hypothesized that a surgical stimulus and the administration of these drugs would modify the PTAm, and that a correlation would be found between PTAm, HR, and MAP.

## 2. Materials and Methods

### 2.1. Animals

Twenty client-owned healthy adult horses were enrolled in this prospective, observational, clinical study. All ASA I or II horses received at the Veterinary Teaching Hospital, University of Córdoba, in a period of 5 months, whose owner accepted to participate, were included in the study. Surgeries included 15 arthroscopies, 1 tenoscopy, 3 castrations, and 1 umbilical hernia repair.

Horses were determined to be healthy based on physical examination, a complete blood count, and a serum biochemistry panel. The study was approved by the Ethical Committee of Animal Welfare of the Veterinary Teaching Hospital, University of Córdoba (CEBAHCV60/2018). All procedures were conducted in compliance with the ethical principles of good practice in animal experimentation.

### 2.2. Anesthesia and Instrumentation

Food was withheld 8 h prior to elective surgery. An intravenous (IV) catheter was placed in the right jugular vein the morning of the procedure. All animals received phenylbutazone 4.4 mg/kg IV (Butasyl^®^, Zoetis, Madrid, Spain), sodium benzyl penicillin 22,000 IU/kg IV (Penilevel^®^, Laboratories ERN SA, Barcelona, Spain), and gentamicin 6.6 mg/kg IV (Genta-Equine^®^, Dechra, Barcelona, Spain) 30 min prior to the premedication. Premedication consisted of xylazine 1.5 mg/kg IV (Xilagesic^®^, Laboratories Calier SA, Barcelona, Spain). Ten minutes later, anesthesia was induced with diazepam 0.1 mg/kg IV (Valium^®^, Roche Farma SA, Madrid, Spain), and then ketamine 2.7 mg/kg IV (Imalgene^®^, Merial, Toulouse, France), 1 min apart. Horses were then intubated with an appropriate endotracheal tube using a blind technique, hoisted onto the surgical table, and positioned in dorsal recumbency. Horses were attached to a large animal anesthesia breathing circuit (LDS 3000 Large Animal Anesthesia Machine, Surgivet, Smith Medical, Barcelona, Spain). Anesthesia was maintained with end-tidal isoflurane of 1.3–1.6% (IsoVet^®^, Piramal Healthcare UK Limited, Northumberland, UK) in 100% oxygen. The depth of anesthesia was assessed by a single anesthetist based on palpebral reflex, the presence or absence of nystagmus, movement, and neck muscle relaxation. The objective was to maintain a surgical plane of anesthesia in which the animal had no or light palpebral reflex and did not respond to the surgical stimulation. Eucapnia (end-tidal CO_2_ 35–45 mmHg) was maintained using volume-controlled mechanical ventilation, beginning upon connection to the anesthesia machine. The ventilator was set up with a tidal volume of 10 mL/kg, peak pressure of 20 mmHg, respiratory rate of 8–10 breaths/minute, and I:E ratio of 1:2. All horses received a Lactated Ringer’s solution infusion at 5 mL/kg/h IV (Lactato de Ringer^®^, Braun Medical SA, Barcelona, Spain) and morphine at 0.2 mg/kg IV (Morfina^®^, Braun Medical SA, Barcelona, Spain).

The facial artery was catheterized using a 20-gauge cannula (VasoVet^®^, Braun Medical SA, Barcelona, Spain) for invasive measurement of blood pressure. The arterial cannula was connected to a transducer positioned at the sternum level and zeroed. If an MAP of ≤62 mmHg was observed, dobutamine (Dobutamina^®^, Inibsa Hospital SLU, Barcelona, Spain) at 0.25 μg/kg/min IV was administered and increased by 0.25 μg/kg/min every 5 min until the MAP reached target values between 70 and 80 mmHg. If the MAP increased above 80 mmHg, the dobutamine CRI was reduced using similar proportions and stopped if the MAP reached the target values. Ketamine at 0.5 mg/kg IV was administered if a sudden increase in the palpebral reflex, nystagmus, or spontaneous ventilation was observed.

### 2.3. Monitoring

Standard respiratory and cardiovascular variables were monitored over the intraoperative period every 5 min using a pre-calibrated multiparametric monitor (Datex Ohmeda, Multiparameter Monitor, GE Healthcare, Helsinki, Finland). Furthermore, HR, invasive MAP, and PTAm were recorded every minute, and values were statistically analyzed and compared with the previous minute (baseline) and at different time points after the events of interest (see below). The three electrodes of the PTA monitor used to register the electrocardiogram were applied with flattened crocodile clips in the following manner: the red and yellow electrodes were positioned similarly to the same colors of lead II, while the black electrode was placed under the right forelimb, opposite to the green electrode of lead II.

### 2.4. Events of Interest

The HR, MAP, and PTAm were recorded before and after the ‘events’ of interest below.

Incision event—The nociceptive surgical stimulus was considered as the first skin incision for soft tissue surgeries or the first skin incision and the introduction of the scope for arthroscopy (incision event). Therefore, the three variables (HR, MAP, and PTAm) were recorded before and at 1, 3, and 5 min after the incision event.

Morphine event—One minute after the data above were registered, morphine hydrochloride at 0.2 mg/kg IV was administered (morphine event) to all horses, and the three variables were recorded 1 min before and 10 min after its administration.

Ketamine event—In the case of administration of ketamine, the HR, MAP, and PTAm were registered 1 min before (retrospectively) and 3 and 5 min after ketamine administration.

Dobutamine event—In the case of dobutamine administration, the HR, MAP, and PTAm were registered 1 min before (retrospectively) and 5 min after starting/increasing dobutamine CRI.

PTAm, HR, and MAP events—Changes in PTAm, HR, and MAP were also considered events. If a decrease in PTAm of ≥20% (PTA event), an increase in HR of ≥10% (HR event), or an increase in MAP of ≥20% (MAP event) when compared with the previous minute was observed, the retrospective PTAm, HR, and MAP value from 1 min before and 1, 3, and 5 min after the event were registered. However, increases in the MAP or HR during the administration of dobutamine were not considered as a MAP or HR event. In order to avoid confusion with a MAP increase due to dobutamine administration, an MAP event was only considered if the MAP value was stable during the last 10 min before increasing to ≥20% of its baseline value. Recordings were made by one observer (PRL), whereas general anesthesia was provided by a second observer (MMG). The latter individual decided if ketamine and dobutamine were required during the procedure without the input of the former.

### 2.5. Data Analysis

A sample size of 20 animals was chosen (G*Power^®^, v. 3.1.9.2., Düsseldorf, Germany), based on a significance level of 0.05 and a power level of 0.95, to identify a decrease of 15% from a PTAm value of 50, a standard deviation of 12, and an effect size of 0.6, considering a 10% loss of follow up.

Data were excluded from analysis according to the following scenarios: (1) if morphine, ketamine, or dobutamine events occurred at the same time; and (2) if the PTA had poor signal quality (energy was <0.05 or >2.5) during any event of interest.

The distribution of PTAm, HR, and MAP was confirmed to be non-normal using a Shapiro–Wilk test. For each event, baseline values of PTAm, HR, and MAP were compared to data after the event, using a Friedman’s test with a Bonferroni post-hoc test. A Wilcoxon’s test was performed when significant differences were observed. Data were expressed as median (25–75th percentile range). A Spearman’s correlation was performed for PTAm, HR, and MAP using the incision event data. A *p*-value of <0.05 was considered statistically significant (IBM^®^ SPSS^®^ Statistics for Windows, version 25.0, IBM Co., Armonk, NY, USA).

## 3. Results

The horses included in this study were 17 males and 3 females, between 1 and 11 years old and 529.5 ± 68.9 kg.

No complications occurred during the study. No event was detected simultaneously with other events or during poor quality signal; thus, no event was excluded. A minimum 60 min interval elapsed between the administration of xylazine (sedation) and the incision event. In total, 15 ketamine events and 28 dobutamine events were recorded (Table 1).

The PTAm and HR showed a weak Spearman’s correlation (*r* = 0.25; *p* = 0.02), while no correlation was found between PTAm and MAP (*r* = 0.08; *p* = 0.5) at the incision event.

A decrease in the MAP was observed 10 min after administration of morphine (−6 mmHg: 95% CI −2/−10 mmHg; *p* = 0.009). When ketamine was administered, the PTA decreased after 3 min (−16: 95% CI −2.5/−27.5; *p* = 0.04), and the MAP decreased after 5 min (−5.5 mmHg: 95% CI −12/−2 mmHg; *p* = 0.01). The MAP increased 5 min after the administration of dobutamine was started (10 mmHg: 95% CI 7–14 mmHg; *p* = 0.001). The maximum CRI dose of dobutamine administered was 0.5 μg/kg/min.

Seven PTAm events were recorded (−24: 95% CI −29/−20; *p* = 0.02). No changes in PTAm were found 3 or 5 min after the PTAm event. Three MAP and two HR events were identified; data were not analyzed due to the low number of events.

## 4. Discussion

Overall, the present study showed that PTAm only decreased after the administration of ketamine, but not after the administration of morphine or dobutamine, or after the incision event. Changes in ANS activity were more commonly observed using PTAm rather than HR or MAP.

The administration of opioids may stimulate the central nervous system in horses [17] due to a sympathomimetic effect, as previously described [20]; this is contrary to the depression effect seen in most species. Therefore, there is an interest to know how opioids affect ANS activity and influence PTAm. In humans, it has been reported that fentanyl increases vagal tone and decreases sympathetic nervous system activity [21], and remifentanil increases parasympathetic tone activity, observed as an increase in ANI [1]. Similarly, it has been shown that fentanyl CRI increases parasympathetic tone activity by increasing PTAm in anesthetized horses [14]. The use of morphine has been poorly documented, but is suggested to alter HRV in humans [22]. In the present study, morphine was administered after the incision event to increase the possibility of recording an increase in the ANS due to surgical stimulation. The MAP decreased 10 min after morphine administration. In equine anesthesia, decreases in blood pressure may be due to dorsal recumbency [23], the use of isoflurane [24], and/or the use of mechanical ventilation [25]. Decreases in MAP could also be a result of decreased nociception. In this case, a decrease in sympathetic activity could be expected to register an increase in PTAm [2], which did not occur. It could be due to the morphine dose used in this study. These differences may also be explained by the different distribution, type, and number of opioid receptors, which are species-dependent [26], and receptor polymorphism [27].

Dobutamine is an agonist of β-adrenergic receptors and may increase cardiac contractility and automaticity [19]. The drug is commonly used in equine anesthesia for blood pressure support [18] and the effects of dobutamine on PTA values could be a potential source of bias when assessing intraoperative nociception. When doses from 2 to 10 μg/kg/min were administered to horses undergoing colic surgeries, the PTAm decreased significantly by 12.9% (*p* = 0.03) at 5 min after starting dobutamine CRI. However, similar significant changes in PTAm were not observed in non-colic surgeries [9]. In our study, the PTAm value was not affected by the administration of dobutamine, but the maximum dose was 0.5 μg/kg/min. Indeed, dobutamine increased MAP significantly during surgery [9], as it did in our study. These results could support the use of PTAm to assess ANS activity during elective surgeries even when dobutamine is being administered for the treatment of hypotension. Further investigations might be needed in different scenarios.

Ketamine produces increases in sympathetic tone activity that could decrease PTA values [3]. In our study, the PTAm decreased significantly 3 min after the administration of ketamine. This result is inconsistent with a previous study in which the administration of ketamine after intubation did not change ANI in men [15]. In our study, horses received ketamine when an increased palpebral reflex, the presence of nystagmus, or spontaneous ventilation was observed. Changes in HRV, and therefore in PTAm, may not have occurred before the observation of the clinical signs used for the administration of ketamine. Therefore, one could not rule out whether changes in PTAm values were due to a delayed response to intraoperative nociception or the administration of ketamine. In any case, no change in PTAm was registered 5 min after administration of the ketamine in regards to baseline values. Changes in the PTAm during this period (i.e., light depth of anesthesia) should be carefully used to assess nociception, although the sympathetic effect of ketamine at the administered dose would last for a short period as suggested in humans [15]. Similar results were found in anesthetized horses with ketamine administered under similar conditions to our study, and they could not exclude the sympathomimetic effect of ketamine [14]. The decrease in MAP 5 min after ketamine administration could be due to the analgesic effect of ketamine [28] or to the deeper level of anesthesia attained [29].

Autonomic nervous system activity is difficult to assess during the intraoperative period, considering that the drugs used, the surgical maneuvers, and other factors could influence ANS balance [1]. No significant changes in PTAm, HR, or MAP, or correlation between variables were found during the incision event or nociceptive surgical stimulus; this contrasts with the study by Mansour et al. in dogs [7]. In the latter, an inverse correlation was observed between PTAm and HR, and between PTAm and systolic arterial blood pressure after surgical incision. Similar to our study, Mansour et al. [9] did not find changes in PTAm after cutaneous incision. Some studies have shown that ANI measurements correlate well with nociceptive stimuli [30,31] and predictions of cardiovascular increments [6]. However, the results are controversial since ANI has also shown little predictive value for cardiovascular changes in another study [32]. The PTAm previously demonstrated a correct assessment of nociceptive stimuli in dogs under isoflurane and sevoflurane anesthesia [7,10]. The end-tidal isoflurane in our study was consistently maintained between 1 and 1.23 times the isoflurane minimum alveolar concentration (MAC) in horses. Maybe it would have been more appropriate to evaluate the response of the ANS at a light depth of anesthesia to avoid any interference due to a deep anesthesia plane, but this was not possible due to the clinical characteristics of the study.

The PTAm could identify changes produced by sympathetic tone activation on more occasions (seven occasions) than by an increase in HR (two occasions) or MAP (three occasions). Similarly, in humans, ANI decreased on 95 occasions, but HR increased only on 1 occasion and systolic blood pressure on 14 occasions in a group of 30 patients [32]. Since the PTAm was significantly decreased 1 min after the PTAm event only, it could indicate a brief activation of the sympathetic activity without any further changes in the cardiovascular variables. Furthermore, it has been shown that PTAm changes do not necessarily coincide with cardiovascular changes [10], even when the PTAm showed an acceptable performance to predict an MAP decrease of >10% within 5 min in horses [9].

Agonists of α2-adrenergic receptors can modify sympathovagal balance [33]. Xylazine was used for its shorter half-life (31.4 ± 8.9 min) and duration of action in horses [34] to avoid bias in the study results. However, it cannot be totally assured that xylazine was completely metabolized at the time of incision. Dogs receiving morphine plus medetomidine at premedication showed an increase in blood pressure without a drop in the PTAm during the incision [8]. In the same way, no significant variation in the PTAm was found during surgical incision in horses premedicated with xylazine and anesthetized with sevoflurane undergoing elective or colic surgery [9].

Phenylbutazone, a COX-1 selective nonsteroidal anti-inflammatory drug [35], was administered prior to general anesthesia, which could have influenced intraoperative nociception. It could be a limitation, but since it was a clinical study and considering the welfare of the animals, it was decided to administer phenylbutazone because morphine was planned to be injected after the incision.

A limitation of our study was the lack of validation of the PTA monitor in horses, although the algorithm of the PTA monitor specially designed for use in horses was used in this study. The monitor was used to evaluate the PTAm in response to the HRV without using the PTAm value for nociception evaluation. Another limitation might be the lack of ANS changes during surgical incision. This could indicate adequate intraoperative anesthetic depth and antinociception, or that skin incision was not a supramaximal nociceptive stimulus that would evoke changes in ANS activity during general anesthesia in horses. Incision has also been considered a nociceptive stimulus that decreases PTAm in recent clinical studies in dogs [7,8], but no changes in the PTAm value were shown in horses [9]. Nevertheless, our objective was to evaluate changes in ANS under clinical conditions. Another intrinsic limitation was that the events of interest might be influenced by drugs and surgical interventions. Moreover, the results could be affected by the pre-established time points after each event. Therefore, the instantaneous PTA has been used in dogs to determine nociception [10] since it changes quicker than PTAm. However, the same study determined that changes in PTAm occurred around 60 s after the nociceptive stimulus. The pre-established time points have been fixed considering the pharmacokinetics of the drugs used [36,37].

## 5. Conclusions

No change in ANS activity was observed as PTAm changes after a surgical nociceptive stimulus. Further, the absence of correlations between PTAm and HR or MAP may preclude the use of PTAm to detect sympathetic activation during surgery in horses under the study conditions. However, PTAm changed on more occasions than HR or MAP did, which suggests an earlier detection of ANS activity by the PTA monitor. Only ketamine affected ANS activity by decreasing PTAm under the study conditions. Further studies should be performed to determine how other drugs can affect ANS balance using different cut-offs, time points, and surgical stimuli in anesthetized horses.

## Figures and Tables

**Table 1:** Median (25th–75th percentile range) heart rate (HR), mean parasympathetic tone activity (PTAm), and mean arterial pressure (MAP).

Variable	Baseline	1 min	3 min	5 min	10 min
Incision event					
HR (beats/min)	36 (35–39)	36 (34–38)	37 (36–39)	37(36–39)	-
PTAm (adimensional)	82 (71–90)	79 (63–89)	81 (68–93)	86 (76–94)	-
MAP (mmHg)	76 (69–86)	71 (68–79)	70 (67–78)	71 (64–79)	-
Morphine event					
HR (beats/min)	37 (36–39)	-	-	-	37 (35–39)
PTAm (adimensional)	83 (71–92)	-	-	-	85 (70–94)
MAP (mmHg)	73 (66–80)	-	-	-	67 (60–77) *
Dobutamine event					
HR (beats/min)	38 (35–39)	-	-	38 (34–41)	-
PTAm (adimensional)	73 (59–91)	-	-	67 (61–78)	-
MAP (mmHg)	55 (48–60)	-	-	65 (61–73) *	-
Ketamine event					
HR (beats/min)	41 (34–45)	-	38 (34–45)	39 (34–44)	-
PTAm (adimensional)	71 (59–86)	-	56 (44–65) *	65 (60–76)	-
MAP (mmHg)	78 (67–90)	-	73 (67–85)	69 (67–85) *	-
PTAm event					
HR (beats/min)	37 (34–43)	39 (34–45)	41 (36–45)	42 (36–44)	-
PTAm (adimensional)	86 (80–92)	61 (59–70) *	68 (54–79)	69 (53–81)	-
MAP (mmHg)	86 (76–89)	83 (70–86)	81 (69–86)	83 (67–85)	-

Incision event (*n* = 20): data from before the incision (baseline) and 1, 3, and 5 min after the incision. Morphine event (*n* = 20): data from before the administration of morphine (baseline) and 10 min after the administration of morphine. Dobutamine event (*n* = 28): data from before (baseline) and 5 min after a continuous rate infusion of dobutamine was started or increased. Ketamine event (*n* = 15): data from before (baseline) and 3 and 5 min after the administration of ketamine. PTAm event (*n* = 7): data from before (baseline) and 1, 3, and 5 min after a decrease in mean parasympathetic tone activity (PTAm) ≥ 20% of the previous value was identified. Baseline data were registered 1 min before the event. * Statistically different from baseline (*p* < 0.05). - Not applicable.

## Data Availability

The data presented in this study are available on request from the corresponding author. The data are not publicly available due to confidential identification of the animals.
